# Primary Esophageal Tuberculosis With Dysphagia

**DOI:** 10.7759/cureus.16236

**Published:** 2021-07-07

**Authors:** Mukesh S Paudel, Sandesh R Parajuli, Bidisha Baral, Puskar Poudel, Ishan Dhungana

**Affiliations:** 1 Gastroenterology, National Academy of Medical Sciences, Kathmandu, NPL; 2 Internal Medicine, Nishtar Medical University, Multan, PAK; 3 Medicine, National Tuberculosis Control Centre, Kathmandu, NPL; 4 Internal Medicine, Kathmandu University, Kathmandu, NPL; 5 Pathology, B.P. Koirala Memorial Cancer Hospital, Bharatpur, NPL

**Keywords:** esophageal tuberculosis, endoscopy, dysphagia, caseous necrosis, granulomas

## Abstract

Tuberculosis is a common infection caused by *Mycobacterium tuberculosis. *Tuberculosis can affect many organ systems of the human body including the gastrointestinal tract. Esophageal involvement of tuberculosis is however rare.

A 60 years old male from Nepal with an occupational history suggestive of exposure to tuberculosis presented with dysphagia. He did not have any other complaints and his physical examination was unremarkable. An upper gastrointestinal endoscopic examination revealed an esophageal ulcer at 25 cm from incisors. Biopsy from the edge of the ulcer revealed granulomas with central caseous necrosis. A computed tomography scan of the chest and abdomen did not reveal additional lesions. Considering the higher prevalence of tuberculosis in the geographical area, he was started on an empirical antitubercular regimen. His dysphagia subsided within two weeks of starting therapy. A repeat upper gastrointestinal examination at six months of therapy revealed complete healing of the esophageal lesion.

In this case report, we review the symptomatology, diagnosis, and treatment of esophageal tuberculosis.

## Introduction

Tuberculosis (TB) is an infectious disease caused by *Mycobacterium tuberculosis*. In 2019, approximately 10 million people developed TB all over the world, and there were an estimated 1.2 million TB deaths among HIV-negative people [1]. The prevalence of TB in Nepal is 416 per 100,000 population.

TB can involve any part of the body except nails and hair and predominantly affects the respiratory system. Lymph node TB followed by tubercular pleural effusion are the commonest types of extrapulmonary TB [2]. Gastrointestinal (GI) TB is a rare entity and mostly involves the peritoneum and distal ileum.

GI system involvement is seen in 11% of patients with extrapulmonary TB. Involvement of the abdomen may occur in the form of GI tract involvement, mesenteric lymph nodes involvement, or involvement of peritoneum or solid organs [3]. Involvement of the GI tract occurs through ingestion of infected sputum or hematogenous spread from primary pulmonary TB [4]. Esophageal involvement of TB is one of the rarest manifestations of extrapulmonary TB with an incidence of <0.2% of all TB cases [5]. TB of the esophagus can be primary or secondary and is more commonly seen in patients with acquired immunodeficiency syndrome (AIDS) [6]. In a primary esophageal TB, no other sites of concurrent tubercular lesions can be detected. However, concomitant pulmonary TB is seen in less than 50% of the patients [7].

We describe a case of primary esophageal TB without immunodeficiency presenting with dysphagia.

## Case presentation

A 60-year-old male from Nepal presented to the hospital with new-onset difficulty while swallowing for fifteen days. The dysphagia was more with solids than liquids. There were no other cardinal features of TB like fever, night sweats and weight loss. The patient was a non-smoker, non-alcoholic and with no known immunodeficiency. His HIV-1 and HIV-2 serology were negative. He had worked as a helper in a DOTS (Directly Observed Treatment, Short-course) center of a community hospital for 15 years. On physical examination, the abdomen was soft without any palpable mass and cervical lymph nodes were non-palpable.

**Figure 1:**
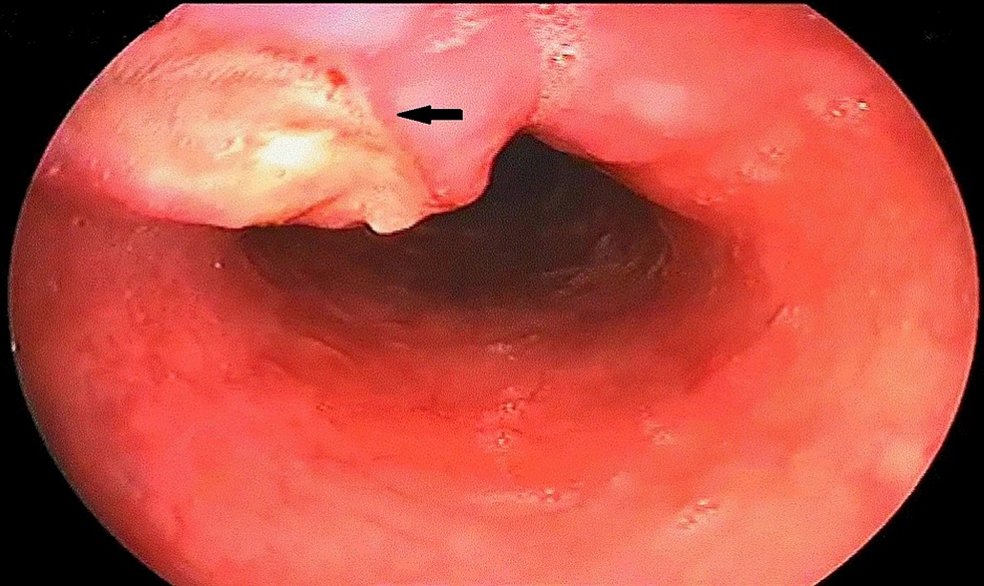
Upper gastrointestinal endoscopy revealed an ulcer in the esophagus 1.5×1.5 cm in size with a whitish base and no bleeding (black arrow).

He then underwent an upper GI endoscopic evaluation, which showed an ulcer at 25 cm from the incisor's edge. The ulcer was 1.5×1.5 cm in size with a whitish base and no bleeding (Figure 1).

**Figure 2:**
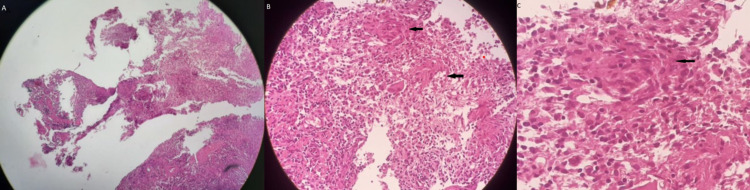
Histopathology of the ulcer edge showed (A) marked chronic inflammatory cells infiltrations mixed with few ill-defined granulomas, giant cells, and foci of caseous necrosis (black arrows in B and C).

An endoscopic biopsy of the lesion was taken. Histopathological evaluation revealed multiple bits of tissue lined focally by keratinized stratified squamous epithelium and underlying stroma showed marked chronic inflammatory cells infiltrations admixed with few ill-defined granulomas, giant cells, and foci of caseous necrosis, features suggestive of the chronic necrotizing granulomatous lesion. This presence of a chronic granulomatous lesion with central caseous necrosis was highly suspicious of TB (Figures 2A-2C).

Gastric biopsies for *Helicobacter pylori* were negative. His lab reports were significant for elevated ESR (20 mm in the first hour) and the Mantoux test, which showed 20 mm induration. Computed Tomography (CT) of the chest and abdomen revealed no other significant findings. Therefore, a provisional diagnosis of primary esophageal TB was made and was started on a protocol-driven four-drug anti-tubercular treatment with rifampicin, isoniazid, pyrazinamide, and ethambutol.

**Figure 3:**
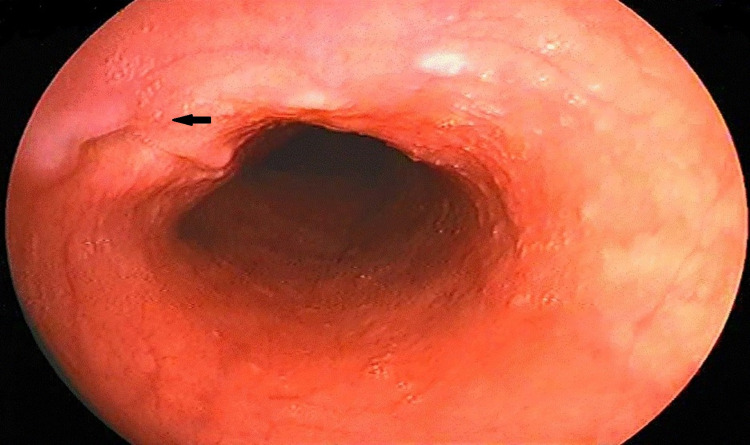
Endoscopic image of the healed esophageal ulcer after six months completion of antitubercular therapy (black arrow).

The patient was followed up initially every two weeks for the first two months and then two months till his completion of anti-tubercular therapy. There were no significant side effects noted with the treatment. His dysphagia gradually subsided after two weeks of treatment. An upper GI endoscopic examination was repeated after six months of starting therapy, which showed a healed esophageal ulcer (Figure 3).

## Discussion

In 2020, the COVID-19 pandemic dislodged TB from the top infectious disease cause of mortality globally [1]. In Nepal, in 2018, about 69,000 people were living with TB, but only 32,474 cases were reported in the National Tuberculosis Program [8]. While most of the cases of TB are pulmonary; extrapulmonary TB (EPTB) accounts for above one-fifth of the global burden of the disease [9]. Of the various EPTB sites, GI TB is the sixth most common site for the disease but very few of them are in the esophagus [7]. Esophageal TB mostly occurs secondary to direct extension from nearby structures like mediastinal lymph nodes or pulmonary sites [10]. TB of the esophagus usually involves the middle third of the organ [11]. Our patient did not have lung or mediastinal lymph node involvement of TB.

It is rare for a patient with esophageal TB to not present with dysphagia or odynophagia. Our patient also had presented with new-onset dysphagia. Depth of the esophageal involvement determines the symptoms. The predominant symptoms may vary depending on inflammation of the mucosa, formation of ulcers or exophytic lesions, and stenosis. The lesion in the esophagus can also complicate fibrosis or stricture due to chronic inflammation, diverticula formation, and rarely tracheoesophageal fistula [12]. Dysphagia is the most common presentation of an esophageal illness including 90% of esophageal TB and its evaluation always begins with an upper GI endoscopy [13].

The exposure history of the patient to TB and local prevalence of the disease contribute to a high index of suspicion in symptomatic patients. Contact with patients with TB increase the suspicion of active TB. The extent of GI TB is usually evaluated by CT of the chest and abdomen [14]. In patients with esophageal TB, the radiological features are not specific and the presence of granulomas with central caseous necrosis in a patient with a history of contact can be considered diagnostic. Other supporting tests like tuberculin skin tests can be done. The stasis of ingested sputum or infected milk products is believed to be one of the causes of GI TB [15]. In our patient, the location of a lesion at the level of the natural constriction of the esophagus by the aortic arch supports this theory. The other modality for the diagnosis of TB is PCR [16], which was not done in our patient. In our case, the biopsy report of necrotizing granuloma and prevalence of TB in our part of the world led us to the provisional diagnosis of TB in this case. This was further supported by a complete response with antitubercular therapy.

Standard anti-tubercular treatment is the treatment of choice for GI TB. It is a four-drug combination comprising isoniazid, rifampicin, pyrazinamide, and ethambutol. A six-month therapy is sufficient for GI TB [17]. Surgery in GI TB is reserved for non-resolving intestinal obstruction, abscess, perforation, or fistula formation [5]. Even tubercular strictures significantly resolve with antitubercular drugs alone [18].

## Conclusions

In parts of the world where TB is endemic, an unusual presentation warrants suspicion of TB. TB of the esophagus is rare. Patients with TB esophagus generally present with dysphagia. A provisional diagnosis of TB esophagus can be made if granulomas with caseous necrosis are visualized during a histopathological examination of the lesions. Polymerase chain reaction (PCR) for detection of mycobacterial DNA and cultures of the materials for detection of mycobacteria can be done. In cases, where such investigating modalities are not available, an empirical trial of anti-tubercular drugs can be undertaken. We also recommend taking into consideration of national and regional guidelines for the detection and treatment of TB.

## References

[1] Chakaya J, Khan M, Ntoumi F (2021). Global Tuberculosis Report 2020 - reflections on the global TB burden, treatment and prevention efforts [PREPRINT]. Int J Infect Dis.

[2] Paudel M, Kafle A, Chhetri BK, Dhungana SP, Poudel A (2013). Characteristics of patients with tuberculous pleural effusion in rural Nepal. J Lumbini Med Coll.

[3] Rathi P, Gambhire P (2016). Abdominal tuberculosis. J Assoc Physicians India.

[4] Welzel TM, Kawan T, Bohle W, Richter GM, Bosse A, Zoller WG (2010). An unusual cause of dysphagia: esophageal tuberculosis. J Gastrointestin Liver Dis.

[5] Debi U, Ravisankar V, Prasad KK, Sinha SK, Sharma AK (2014). Abdominal tuberculosis of the gastrointestinal tract: revisited. World J Gastroenterol.

[6] Momin RN, Chong VH (2012). Oesophageal tuberculosis: rare but not to be forgotten. Singapore Med J.

[7] Sheer TA, Coyle WJ (2003). Gastrointestinal tuberculosis. Curr Gastroenterol Rep.

[8] Gautam N, Karki RR, Khanam R (2021). Knowledge on tuberculosis and utilization of DOTS service by tuberculosis patients in Lalitpur District, Nepal. PLoS One.

[9] Kulchavenya E (2014). Extrapulmonary tuberculosis: are statistical reports accurate?. Ther Adv Infect Dis.

[10] Leung VK, Chan WH, Chow TL, Luk IS, Chau TN, Loke TK (2006). Oesophageal tuberculosis mimicking oesophageal carcinoma. Hong Kong Med J.

[11] Huang YK, Wu YC, Liu YH, Liu HP (2004). Esophageal tuberculosis mimicking submucosal tumor. Interact Cardiovasc Thorac Surg.

[12] Mbiine R, Kabuye R, Lekuya HM, Manyillirah W (2018). Tuberculosis as a primary cause of oesophageal stricture: a case report. J Cardiothorac Surg.

[13] Triggs J, Pandolfino J (2019). Recent advances in dysphagia management. F1000Res.

[14] Ladumor H, Al-Mohannadi S, Ameerudeen FS, Ladumor S, Fadl S (2021). TB or not TB: a comprehensive review of imaging manifestations of abdominal tuberculosis and its mimics. Clin Imaging.

[15] Kasulke RJ, Anderson WJ, Gupta SK, Gliedman ML (1981). Primary tuberculous enterocolitis. Report of three cases and review of the literature. Arch Surg.

[16] Shad S, Rai AA, Soomro GB, Luck NH (2018). Tuberculous paraspinal abscess invading esophagus: a rare cause of dysphagia. J Coll Physicians Surg Pak.

[17] Mandavdhare HS, Singh H, Dutta U, Sharma V (2019). A real-world experience with 6 months of antitubercular therapy in abdominal tuberculosis. JGH Open.

[18] Anand BS, Nanda R, Sachdev GK (1988). Response of tuberculous stricture to antituberculous treatment. Gut.

